# The “autonomy” of developing countries in the Olympic Movement: Assessing the fate of sports governance transplants in the Global South

**DOI:** 10.3389/fspor.2022.972717

**Published:** 2022-12-01

**Authors:** Borja García, Henk Erik Meier

**Affiliations:** ^1^School of Sport, Exercise and Health Sciences, Loughborough University, Loughborough, United Kingdom; ^2^Institute of Sport and Exercise Sciences, Münster University, Münster, Germany

**Keywords:** transnational private governance, International Olympic Committee (IOC), sports autonomy, governance transplants, Global South, Guatemala, Botswana, Sri Lanka

## Abstract

**Introduction:**

The International Olympic Committee (IOC) imposes very specific ideas on sports governance, more precisely on sports autonomy, on countries joining the Olympic Movement. Given that the idea of sports autonomy originated in the Global North, this article introduces the concept of governance transplants to evaluate the impact that being part of the Olympic Movement has on domestic sports governance in Global South developing countries. The article explores the extent to which the IOC is successful in implementing the norms and regulations on sports autonomy as a governance transplant at the national level in countries from the Global South that are part of the Olympic Movement.

**Methods:**

The article employs a comparative qualitative case study research design that explores the relations of the IOC, National Olympic Committees (NOCs), and national governments in Botswana, Guatemala and Sri Lanka. Research relies on a mix of document analyses and expert semi-structured interviews conducted during field trips to those countries. Interviews were transcribed and analyzed by means of thematic analysis. The analyses focus on domestic policies and contexts, formal and institutional compliance with sports autonomy, provision of public funding, and participation in national sport policy-making.

**Results and discussion:**

Findings suggest that national structures and legacies have an impact on the way in which the autonomy of sport, as the governance transplant, is translated in those three countries. Although national governments enjoy some agency in “translating” governance transplants, results also suggest that misfits and tensions persist between governmental and sport stakeholders at the national and international level. Such misfits might force the IOC, as a private transnational regulator, to adopt a more pragmatic view on the enforcing of its governance transplants. The results are of relevance to existing discussions on global sports governance and debates as to whether the countries in the Global North might be able to impose their views and their governance transplants if the Global South gets a greater say in transnational sports governance.

## Introduction

The interest of developing countries in being recognized by the International Olympic Committee (IOC) and being part of the Olympic Games has been well-documented. Joining the Olympic Movement “is one of the “signs of statehood” that gains them [countries] recognition from the global community” [(1), p. 1005]. In particular post-colonial or smaller countries, which might lack “historical or other contemporary sources of national recognition,” use sport often to present the nation at the global stage [(2), p. 100]. Moreover, according to Houlihan and Zheng (3), being part of the Olympic Movement gives small states visibility in the international arena, because participant countries are granted an equal symbolic status. Finally, being part of the Olympic Movement can have a positive impact in sports development of developing countries due to IOC funding (1).

However, when developing countries (or any country, for that matter) become part of the Olympic Movement, they enter a very particular and well-defined governance structure with a set of core policies and beliefs. Indeed, countries join the Olympic Movement when their National Olympic Committee (NOC) is formally recognized by the IOC. And it is the IOC who decides on the criteria for such recognition. Countries and their sport systems also have to recognize and respect the provisions of the Olympic Charter and the authority of the IOC as the highest governing body of the Olympic Movement. The IOC is not only the gatekeeper of this process, but also an agent of policy transfer who partly imposes its vision on the structures that new countries' sport systems need to adopt to join the Olympic Movement.

In that context, the aim of this article is to explore the relationship between the policies of the IOC at the international level and the regulatory structures of sport at the national level in developing countries. The article aims to assess the impact of IOC norms on developing countries, and whether these might have any room for agency in the application and implementation of these norms at national level.

Thus, our first research objective is to assess the extent to which the IOC is able to implement its policies on sports governance in countries in the Global South that are part of the Olympic Movement. We use the terms Global South and Global North to distinguish the generally less prosperous nations of Asia, Africa, and Latin America from the wealthy industrialized nations (including the U.S., Canada, Australia, New Zealand, Japan, and European Union member states). In contrast to alternative notions such as developing countries, the concept of the Global South indicates that these countries share a history of Northern economic and political domination and face persistent power asymmetries. As the Global North is wealthier and more powerful, it can use its control of international organizations to globalize its discourses and to pursue its ideas of developmentalism and teleological progress according to which the Global South has to “catch up” (4). Hence, the article's second objective is to investigate the extent to which countries in the Global South might find it difficult fitting into the governance structures of the Olympic Movement. Our final objective is to evaluate how those countries implement the requirements of the IOC into their national sport systems.

These aims and objectives are relevant because, as Reiche has pointed out [(1), p. 997], research into the participation of emerging countries in the Olympic Movement is not excessively developed. Whereas the benefits have been explored (see above), other consequences, such as the impact on national sports governance and policy, have received less attention.

In order to meet our research objectives, the article focuses on the implementation of a fundamental principle of the Olympic Movement: The autonomy of sport. The autonomy of sport is one of the main pillars of the Olympic Movement's governance; it is defined as the demand that the regulation of sport falls into the exclusive domain of sport governing bodies and that sport bodies do not suffer any political interference from governments or other political actors (5). Thus, sports autonomy seeks to ensure “the preservation of the values of sport, the integrity of competitions, the motivation and participation of volunteers, the education of young people and their contribution to the wellbeing of all, women, men and children, thereby contributing to its credibility and legitimacy” (5, 6). The strategic importance of sports autonomy for the Olympic Movement is demonstrated in the Olympic Charter, which stipulates that one of the missions of the IOC is, indeed, “to preserve the autonomy of sport” [(7), Art. 2.5].

The IOC expects, therefore, countries participating in the Olympics to adhere to its standards of sports autonomy as specified in the IOC's regulation (5, 8). In doing that, the IOC acts as a powerful transnational private regulator because it defines the norms for any actor or stakeholder who wants to join the Olympic Movement or interact with it (9). This debate is even more interesting because, formally, the rules of the IOC are regulations of a private nature and should not affect public sports policy. The Olympic Charter is not an international treaty, nor public regulation. However, as Cafaggi [(10), p. 4–5)] has put it:

While, formally, private regulations are voluntary instruments to which parties are free to subscribe or alternatively, abstain, their adoption has often come to be understood as the precondition for access to global markets or other regulatory spaces. Frequently then, these transnational private regimes are legally voluntary but socially or economically binding.

Compliance with the norms defined by the IOC implies usually amending existing national laws or adopting new ones in order to ensure the structures of the national sports system complies with the requirements of the Olympic Charter. As James and Osborn [(11), p. 99] elaborate, the IOC “does not fulfill its tasks in cooperation with states, but by requiring them to act on its behalf.”

In order to operationalise our analysis, the article introduces the concept of “governance transplants,” which is inspired by the debate about “legal transplants” in comparative legal research [e.g., (12)]. The article conceptualizes the autonomy of sport as a governance transplant; that is to say, a set of rules and norms defined by the IOC that countries joining the Olympic Movement are required to incorporate into their national sports systems. Thus, the article explores, in a nutshell, how successful the IOC might have been on transplanting its governance concept of sports autonomy, and whether (like in traditional transplants) misfits and tension persist.

Methodologically, the article adopts a qualitative comparative case study research design. A comparative case study on “sports autonomy” in three countries from the Global South (Botswana, Guatemala, and Sri Lanka) to explore how national political structures have adapted to and negotiated the requirements of autonomy imposed by the IOC. Data collection is done through analysis of national sports policy documents and semi structured interviews with representatives of sport stakeholders in those countries.

The article proceeds now in four steps. First, we present our analytical framework based on the concept of governance transplants. Second, we discuss our research design and methods. Third, we present the results of the three case studies adopting a comparative approach. Finally, we discuss the implication of our findings.

## Theoretical framework

### Governance transplants as analytical concept

When countries are part of the Olympics, they need to abide by the rules contained in the Olympic Charter. This qualifies as a policy transfer from the IOC to the members of the Olympic Movement. Whereas policy transfer is commonly associated with the transfer of institutions (13), policy transfer can also refer to the transfer of goals, tools, ideas and ideologies as well [see (6, 14, 15)]. In order to emphasize that such policy transfers can challenge domestic political and social systems in a fundamental manner, we introduce the concept of “governance transplants.” The transplant metaphor is borrowed from the notion of legal transplants, which refers to the application of laws and norms designed in a particular legal context to a different environment for which the norms were not designed (12). Comparative legal research claims that a transplanted law must “fit” with the institutional, social and cultural context to which it is transplanted in order to work appropriately (12). This fit, however, is not necessarily easy to achieve since, as historical institutionalist arguments argue, domestic governance arrangements reflect deeply entrenched national policy traditions, which prevent a simple policy transfer (16, 17). Indeed, policy transfer research has stressed that certain policies are not transferable because they are neither ideologically or culturally proximate (13). It is certainly no coincidence that the notion of “governance transplants” has already been used before within the context of comparative corporate governance research. Here, the notion served to indicate that U.S. models of corporate governance cannot be easily transferred to other contexts and that the efficacy of corporate governance models is path-dependent and contingent on local configurations of formal and informal institutions (18, 19). Moreover, scholars have used the notion to emphasize that governance transplants may result in “superficial convergence in form but divergence in function” [(20), p. 880].

Essential to our concept is that governance transplants, such as the autonomy of sport, do not aim to transfer isolated policies, instruments or tools but affect a very fundamental dimension of governance, that is, the distribution of responsibilities between public authorities, private actors and society for producing policy outcomes (in our case, the division of competencies between national governments, on the one hand, and sports federations and NOCs, on the other). Governance transplants can, thus, challenge the incumbent domestic understanding of the role of governments, which is the path-dependent outcome of historical struggles and compromises. Accordingly, a misfit between governance transplants and domestic governance arrangements is likely to occur, and it has the potential to impede compliance and implementation.

Marsh and Sharman (21) emphasize that there is a degree of agency of the receiving countries in policy transfers, which inspired Stone (22) to employ the notion of “policy translation.” Policy translation is often a dynamic, creative and haphazared process, which involves bricolage, experimentalism and learning (22).

Depending on the degree of misfit between the transplants and local preconditions, governance transplants might be modeled and structured (i.e., “translated”) in a way that their impact on domestic governance arrangements is minimized. Therefore, we ask to which extent the countries in the Global South might be able to shape or modify the IOC governance transplant into their sport governance structures or not.

### Sports autonomy as governance transplant

The IOC's key governance transplant that we analyse in this article is, as explained above, sports autonomy. This is a principle of strategic importance for the IOC and the Olympic Movement, as demonstrated not only by its inclusion in the Olympic Charter, [(7), Art. 2.5], but also in the so-called Basic Universal Principles of Good Governance of the Olympic Movement (23). The concept of sports autonomy, originated in the Global North where liberal political ideology forced governments to practice self-restraint in regulating citizens' leisure and where a strong and resourceful civil society could provide sport activities (24). Sports autonomy qualifies as a governance transplant because it restricts state sovereignty and political discretion, and raises serious questions about democratic accountability (25, 26). Moreover, sports autonomy rests on very specific political and societal prerequisites, that is, political self-restraint and a strong civil society.

The IOC implants sports autonomy into national sports systems via the NOCs. The NOCs are located between the IOC and national governments. The NOCs are not governmental bodies but act as the national representative of the IOC within the respective country (11). The Olympic Charter demands the NOCs to “preserve their autonomy and resist all pressures of any kind, including but not limited to political, legal, religious or economic pressures” [(7), Art. 27.6]. However, existing research on NOCs indicates that their autonomy is contested outside the Global North. Chappelet and Kübler-Mabbot (27) claimed that the majority of NOCs is actually government-controlled via funding or political ties [see also: (28)]. We try to demonstrate that the contested state of sports autonomy reflects the substantial misfit between the governance transplant and the receiving national systems. Although national public authorities are forced to formally comply, they “translate” the governance transplant (i.e., the autonomy of sport) according to the logics of domestic sport policy-making.

## Research design and methods

The article adopts a comparative qualitative case study research design, which is the preferred methodological choice of the existing literature on policy transfer that our theoretical framework is built upon (13, 22). This is a logical choice, as case study research design is especially suited for research that (1) studies a phenomenon over a period of time, (2) uses data from different sources, (3) answers research questions of how and why, and (4) it is delimited in terms of geographical scope (29). In our case, we explore the compliance with sports autonomy and the relations between public authorities and NOCs with a longitudinal perspective, and with a clear geographical scope of the chosen country. Our research clearly focuses on exploring the hows and whys of the implementation of sports autonomy as a governance transplant, hence the case study research design is a valid epistemological choice for this research. Finally, our research does indeed collect data from a variety of sources, as we employ both analysis of official policy documents and semi-structured interviews as research methods.

Indeed, as State [(30), p. 1] points out a case study research design “looks for the detail of the interaction with its contexts,” which summarizes very aptly this article's research aims and confirms the suitability of our methodological choice.

The case studies employ the method of structured focused comparison (31) in order to explore not only formal compliance to IOC provisions on sports autonomy, but also the interactions between public authorities and NOCs in the three selected countries.

Our case selection was informed by the following criteria. Countries have to (1) come from the Global South, (2) be part of the Olympic Movement, (3) have a recognized NOC, and (4) show sufficient formal compliance with sports autonomy. In order to fulfill the last point, countries could not have their membership of the IOC suspended because of political interference (i.e., lack of autonomy) at the time the research was done. Moreover, we aimed to select countries with a substantial variety concerning domestic legacies likely to create possible misfits with the governance transplant. Therefore, whereas all countries had to comply with the selection criteria, the research design also aimed for variety in order to enhance the explanatory powers of the study.

Once all the countries meeting the criteria were listed, we proceeded to contact the respective NOCs, whose level of response was variable. The IOC services advised the research team on the NOCs that tended to be more responsive to external requests, and the levels of responsiveness they used to have from NOCs around the world. A final shortlist of 20 countries was done amongst those who responded to the call and could provide the level of information required. This led to the final selection of Botswana, Guatemala and Sri Lanka because these three countries met the sampling criteria, provided the best access to interviewees and documents, and represented a good global spread of different sport, political and social cultures. Decisions on shortlisting and final selection of the three cases were taken exclusively by the research team, without input from the IOC. We acknowledge that this final selection was informed by access, as it was necessary to secure the widest possible access to sources given the nature of the research, and that needs to be listed as a limitation. However, the three countries do meet the pre-stablished sampling criteria and provide a good geographical spread. We would argue that such spread, coupled with the access to participants and documents to provide rich data sources outweighs that limitation. The three countries are small in population terms, characterized by low GDP per capita and by limited success at the Olympics. However, we argue that they show substantial variance in terms of their NOC's operational capacity, as demonstrated in their NOC's budget, share of public spending and size (Table 1).

**Table 1:** Characteristics of the country case studies.

**Indicator**	**Guatemala**	**Botswana**	**Sri Lanka**
Population in million	17.263	2.250	21.670
GDP per Capita in US$	8,413	18,654	13,443
National independence since	1,823	1,966	1,948
NOC recognized in	1,947	1,980	1,937
First participation in the Olympics	1,952 (Helsinki)	1,980 (Moscow)	1,948 (London)
Olympic medals won			
Gold	0	0	0
Silver	1	1	2
Bronze	0	1	0
Medal rank	134	129	123
NOC budget 2017 in thousand US$	9,633.76	1,359.47	404.84
Share of government funding	92.59%	81.26%	5.22%
NOC staff	150	18	14+4
Number of interviewees	14	25	13

World Bank, Olympic database, financial reports of the NOCs and fieldwork notes.

In order to examine how the governance transplant is received in different national contexts, we focus first on *formal/institutional compliance* with the principle of sports autonomy. However, we build on Dolowitz et al. [(32), p. 460] who have emphasized, that policy transfer should not be examined as one-off event but studied as a process “that develops as it enters and works its way through the domestic policymaking setting.” It is also important to realize that the NOCs are institutionalized as permanent “agents of transfer,” which remain active in the transfer process. Therefore, we argue that the outcome of governance transplants is best studied by examining how the transplant has impacted political practices beyond mere formal compliance. Accordingly, the case studies focus also on the *budgeting process for the NOCs*, and *their role in domestic sport policy-making*.

### Sampling and data collection

The data used here come from two main sources: official documents from governments and NOCs, and expert semi-structured interviews. During three field trips to Botswana, Guatemala and Sri Lanka, we were able to collect both documents and interview 51 key informants. Documents included the sports legal regulatory framework of the country, including primary and secondary legislation, as well as any past and existing sport public policy strategy; policy and strategic documents of NOCs in relation to their collaboration with public authorities were also collected and analyzed.

We followed a purposeful sampling strategy to select interviewees [(33), p. 70–71], which included a combination of maximum variation, and snowball sampling [(33), p. 70–71]. Participants were approached and selected because of the information they could provide about the relationship between the NOC and public authorities in the country. Sampling, therefore, was designed to recruit participants including both NOC and government representatives, but also representatives of sports federations, athletes and/or coaches, in order to have a wider view from stakeholders; this was also supplemented with a decision to include participants from local media and academics, where possible, that could provide a more independent point of view. Participants from external stakeholders were especially important to contrast and triangulate the versions of the NOCs and the governments. Table 2, lists the final composition of the sample, whereas Table 3 provides information about the interviews that are cited in this article. This research followed a strict protocol for data collection, analysis and storage and management. The research received ethical clearance of Loughborough University Research Ethics Review Sub-Committee under reference number HPSC−2495. As participants were granted the right to anonymity, extreme care has been taken to ensure their identities cannot be traced, as can be seen below.

**Table 2:** Interviews' sample composition.

**Stakeholder category**	**Guatemala**	**Botswana**	**Sri Lanka**	**Total**
NOC	5	5	5	15
Governmental body				
Ministry	1	2	4	7
Sport commission/agency	1	3	N/A	4
Other public authorities	1			1
Sport federations	1	4	1	6
Other sport stakeholders				
Athletes		7	1	8
Coaches		1		1
Other	3			3
Media		2	1	3
Independent academics	1	1	1	3
Total number of interviewees	13	25	13	51

**Table 3:** Interviews cited in the text.

**Interview code**	**Interviewee's position**	**Country**	**Location**	**Date**
Interview 1	Official, Ministry of Youth Empowerment, Sport and Cultural Development	Botswana	Gaborone	28/03/2019
Interview 2	Senior director NOC SL	Sri Lanka	Colombo	23/07/2019
Interview 3	Sports coach	Botswana	Gaborone	01/04/2019
Interview 4	Guatemala NOC senior officer	Guatemala	Ciudad de Guatemala	10/04/2019
Interview 5	Guatemala NOC senior officer	Guatemala	Ciudad de Guatemala	08/04/2019
Interview 6	Top government official, Sri Lanka	Sri Lanka	Colombo	24/07/2019
Interview 7	Botswana National Sports Commission Official	Botswana	Gaborone	28/03/2019
Interview 8	Official, Ministry of Youth Empowerment, Sport and Cultural Development	Botswana	Gaborone	28/03/2019
Interview 9	Former NOC SL board member	Sri Lanka	Colombo	22/07/2019
Interview 10	Advisor to Ministry of Telecommunication, Foreign Employment and Sports	Sri Lanka	Colombo	24/07/2019
Interview 11	Junior administration officer, NOC SL	Sri Lanka	Colombo	24/07/2019
Interview 12	Guatemala NOC board member	Guatemala	Ciudad de Guatemala	08/04/2019
Interview 13	Senior NOC SL official	Sri Lanka	Colombo	22/07/2019
Interview 14	Board member CONADER	Guatemala	Ciudad de Guatemala	10/04/2019
Interview 15	Board member, BNSC	Botswana	Gaborone	28/03/2019
Interview 16	Board member Botswana Table Tennis Association	Botswana	Gaborone	29/03/2019
Interview 17	Board member Botswana NOC	Botswana	Gaborone	27/03/2019
Interview 18	Former senior government official	Sri Lanka	Colombo	23/07/2019

It is necessary to acknowledge that in all three cases senior administrators from the NOCs acted as gatekeepers and facilitators, providing access to the NOC and facilitating local contacts. Whereas, this needs to be acknowledged as a limitation for the sake of openness and transparency, the work of those gatekeepers did not prevent access to any stakeholder or participant that was deemed necessary to ensure as representative a sample as possible. Quite to the contrary, the NOCs were open and facilitated the research team's work during their fieldwork visits. Moreover, the inclusion of independent participants ensured we were able to minimize the impact of this limitation.

Written documents were used to inform process tracing of the NOC-government collaborations that are analyzed in each of the case studies. They were also used to inform the design of the interview guides. Since the main objective of this research was to analyse the collaboration (or lack of) between NOCs and national governments, the design of the interview guides built on the methodological recommendations of the academic literature on collaborative governance (34, 35) to allow for structured focused comparison (31). This body of literature recommends that when comparing cases of collaborative governance, focus should be threefold: (1) The socio-political and institutional context, (2) the drivers and dynamics of the collaborative process, and (3) the process outcomes. The interview guides, therefore, were designed to cover these three areas. The first part of the interview guide aimed at understanding the legal and institutional framework of the country's sports system. This was also vastly informed by the written documents, mostly primary and secondary legislation. The second and third parts of the interview revolved around collaboration between NOCs and the government. This was more open ended, as interviewees were asked to name what they thought were the three main areas of collaboration. Within each one of those areas the questions then addressed the dynamics and outcomes, covering the origin of the initiative, sources of funding and human resources, decision-making and implementation, as well as perceived quality of the outcomes. Interviews were, however, semi-structured in nature, which allowed the research teams to focus on the policy collaborations and the structural design according to the level of seniority, knowledge and expertise of each interviewee.

Interviews were transcribed and analyzed following the well stablished stages suggested by Braun and Clarke [(36), p. 87] for thematic analysis: (1) Familiarizing with the data; (2) Generating initial codes; (3) Searching for themes; (4) Reviewing the themes; (5) Refining and naming the themes. Data gathered in Botswana and Sri Lanka was originally in English and analyzed in that language. Data gathered in Guatemala was originally in Spanish, though. To ensure a strong reliability of the analysis, it was analyzed in the original language (rather than being translated and then analyzed). Coding of the data from the three countries was done by the same member of the research team, who is a native Spanish speaker, as well as a fluent English speaker, for practical reasons but also to ensure a homogenous coding process. In order to provide some structure to the analysis, code generation combined a deductive and inductive approach. Informed by the literature on collaborative governance and our framework on policy transplants, three main areas of interest were pre-defined as codes, namely the institutional and policy context, and the participation of the NOC in policy initiatives. Within this wider framework, the material of each country was inductively coded considering the country's particularities. These coding and theme building decisions were intensely discussed within the research team. In the presentation of the findings below this results in sub-themes that are aligned with each one of the country case studies.

## Translating governance transplants in the Global South

We present now the findings of the case studies. This is done thematically and comparing the three countries under each one of the themes. First, we discuss the domestic policy and historical context in which the case studies are situated. Second, we analyse the legal and regulatory framework in our case studies, as this represents the formal compliance with the governance transplant. The third theme discusses funding of the NOCs and the extent to which this might condition their autonomy, hence shaping the outcome of the governance transplant. The final section analyses the participation of NOCs in national sport policy-making.

### Domestic policy contexts and legacies in the case studies

*Botswana*. The Republic of Botswana gained independence on 30 September 1966. Botswana is a parliamentary democracy and one of the most stable countries in Africa. The Botswana National Olympic Committee (BNOC) was established in 1978 and recognized by the IOC in 1980. In contrast to other African countries, sport and sport development did not figure prominently in government policies until the mid-1990's. Since then, the government started to adopt a much more active role. After the mining of diamonds heavily improved the economic situation, the government became the biggest sponsor of elite and grassroots sports (37).

*Guatemala*. Guatemala is an independent republic since 1847 with an eventful history. Regarding sports, Guatemala was one of the pioneering countries in the diffusion of Olympic sports in Latin America during the 1920's. Moreover, the country had a domestic legacy of sports autonomy because after the Guatemalan revolution of 1944, the first democratic government promoted the autonomy of several social institutions. Hence, in 1945, the Guatemala Autonomous Sports Confederation (CDAG) was formed. Sports autonomy was, however, abolished by the juntas, which ruled between 1954 and 1984.

*Sri Lanka*. Sri Lanka is a country with complex economic, socio-political, religious and ethnic social fabric. The country gained independence from the United Kingdom in 1948 but it was only in 1972 when the country became a republic, adopted its first own constitution and was renamed as the Democratic and Socialist Republic of Sri Lanka. The country suffered a civil war for almost 30 years, ending only in 2009, which served to highly politicize every societal sphere. While the Sri Lanka National Olympic Committee (NOC SL) was founded in 1937, national sport culture is dominated by cricket, a non-Olympic sport.

### Formal and institutional compliance with sports autonomy

#### Botswana: Tensions between the NOC and the National Sports Commission

The legal framework for sport in Botswana consists primarily of the Botswana National Sports Commission Act of 2014. The Act created the Botswana National Sports Commission (BNSC), which reflects the governmental ambition to play a central role in national sport policies. This is not well-received by stakeholders in the sports sector:

[The 2014 Act] is very bad … it is a very bad piece of legislation, because it took over the powers from the national federations in relation to the Sports Council. So previously, we would have seven positions [in the Sports Council Board] that national federations could elect six out of seven. So, it was really a body elected by us, by the sport organizations. But now most of the board members of the BNSC are appointed by the government. The balance of power has completely shifted [with the new 2014 Act], and the federations now only elect two out of the eleven members of the [BNSC] board. And, I feel some of the provisions in the Act also were not favorable to sports, not really. (Interview 16)

The Sports Minister holds wide-ranging powers over the BNSC, in particular with respect to appointing key office holders and terminating a board member's term in office. Moreover, the minister may also give the BNSC board “written directions, of a general or specific nature” (BNSC Act of 2014, Art. 6). The BNSC has a very wide remit as it is entrusted with regulating sport “at all levels,” and with providing “leadership and guidance on sport development” (BNSC Act of 2014, Art. 4). In contrast, the 2014 Act barely mentions the BNOC. Besides the call to cooperate with the BNOC in sport development and participation in international competitions, the Act assigns the BNOC the responsibility “for dealing with Olympic matters” (BNSC Act of 2014, Art. 2). Although Botswanan government officials are quite aware of the IOC's provision on NOC autonomy, there is a tendency to perceive the BNOC as a government agency similar to the BNSC:

It is a complex relation, even somehow overlapping […] In a sense, you can say the BNOC is the sub-set of BNSC. (Interview 8)

So, we do have two agencies there implementing sport policy, we have the National Sports Commission, and we have the National Olympic Committee, they are doing sport development programs on behalf of the Ministry. (Interview 1)

As the BNOC is expanding its activities, the relationship between BNSC and BNOC is becoming rather complex resulting in organizational rivalries. Therefore, both organizations perceived the need to develop a Memorandum of Understanding (MoU):

It was decided that it was a good thing to do because whether you like it or not, when we have two entities that serve sport, that serve to develop sport, that serve to promote sport, it is not good if they go differently and not coming together […]. So let us synchronize the way we do things, we will probably make some savings bringing these committees together, the human capital together, the budgets together […] (Interview 15)

The MoU is a relatively short but ambitious document. The MoU considers merging several structures of both organizations, having joint disciplinary committees and auditing practices. The MoU also mentions the development of a joint national strategy. During fieldwork, it seemed just a matter of weeks having the MoU signed:

With the [Botswana National Sport] commission, in principle we have already agreed to a memorandum of understanding between the two parties, what is outstanding now is for the boards to sign, but otherwise the principle of what and how we are cooperating, that has already been agreed (Interview 17).

However, it took some time for the MoU to be finally adopted in November 2020 (38), which clearly signals the resistance to enhance cooperation between the two institutions, despite the positive words of the interviewees. The delay in reaching this agreement was due to several reasons, including a change in the Sports Ministry and the resistance from the BNSC who feared a potential loss of political control over the sport sector.

#### Guatemala: A very strong regulatory and institutional framework

After the rule of several juntas, the democratic constitution of 1985 reinstated sports autonomy in Guatemala. Article 91 of the Constitution provides the base for the financial autonomy of the Guatemalan National Olympic Committee (GNOC), and Article 92 stipulates sports autonomy [authors' own translation]:

Article 91. On the budgetary allocation for sport. It is the duty of the State to encourage and promote the practice of physical education and sport. In order to ensure that, there shall be a budgetary provision of no <3% of the Ordinary General Budget of the state dedicated to sport.

Article 92. Autonomy of sport. It is recognized and guaranteed the autonomy of federated sport through its governing bodies, *Confederación Deportiva Autónoma de Guatemala* and Guatemala National Olympic Committee, both of which have their own legal personality and their own assets. These organizations are hereby exonerated from paying all type of taxes and administrative fees.

Moreover, Article 170 of National Sports Act of 1997 declares that the Olympic Charter takes precedence over any national legal text. The Act further specifies budget allocation and distribution of competencies in sport policy. Accordingly, GNOC receives an annual allocation of 0.3% of the state budget; this means that 10% of the public sport budget is managed by the GNOC.

The Act pursues a holistic approach for the national sport system and defines clear responsibilities for governmental and non-governmental organizations. The responsibility for elite and professional competitive sport, defined as “the autonomous sphere,” falls into the responsibilities of the autonomous body for federated sport in the country (*Confederación Deportiva Autónoma de Guatemala* – CDAG) and of the GNOC. CDAG is responsible for coordinating and structuring the efforts of national sport federations in athlete identification and development, while GNOC is responsible for preparation and development for high performance sport. At present GNOC and CDAG are two separate organizations as the restrictive legislative framework does not facilitate a merger. However, the two organizations have developed a framework agreement for collaboration and shared services.

#### Sri Lanka: Heavy politization in the sport system

Sport in Sri Lanka is regulated through the Law 25/1973, which also recognized the NOC of Sri Lanka (NOC SL), later amended by the Sports (amendment) Act no. 47 of 1993. The 1973 Act is clearly interventionist reflecting the socialist ideology dominating after the country's independence. The 1973 Sports Law provides ample powers to the government and in particular to the Sports Minister and the Director of Sports, a ministerial position below the minister but with major responsibilities. The Minister of Sport has the power to dissolve a national sport federation, or to replace the elected president and executive committee members with an interim management committee. The government makes regular use of this option, and some interviewees suggested that the government might be trying to control NOC SL “through the back door” (Interview 9). Yet, the government justifies such interventions by financial misconduct within the federations (Interview 6). The 1993 amendment of the 1973 Sports Law included some more comprehensive provisions in relation to the NOC SL, mostly in relation to term limits in office, and the need for the annual accounts to be audited by the Auditor General of Sri Lanka.

The highly interventionist legal framework clearly contradicts IOC stipulations on sports autonomy. Therefore, a legal reform has been a major strategic priority for the IOC and NOC SL, in particular since the government released even tighter regulations for sport associations since 2010 (Interview 2). Two meetings between the IOC and delegations of Sri Lanka government and NOC SL officials in Lausanne resulted in some minor changes in 2016 through ministerial regulations. However, the crucial issue of the power of the Sports Minister to dissolve federations' executive committees and appoint interim committees was not addressed. Governmental efforts to comprehensively reform Sri Lanka sports governance never materialized due to multiple changes of government (Interviews 9, 18). Another “cordial” meeting between IOC and Sri Lanka government officials in Lausanne in June 2019 (Interview 10) also failed to produce results due to yet another change of government following elections (Personal correspondence with NOC senior board member, 07/08/2020). This failed legislative reform is characteristic for sport policy-making in Sri Lanka where a constant change of ministers impedes the development of long-term agendas (39). Recent events in the country that included not only COVID-19 but also major economic, social and political crisis have meant no progress has been made in this respect.

### Provision of public funding

#### Botswana: Heavy governmental dependence

Much like the entire Botswanan sport sector, the BNOC is strongly dependent on government funding:

Yes, we receive money from the government. We receive an administration grant on an annual basis for our running costs, and then we receive a games grant that depends on the years that we have games […]. On a non-games year, the contribution of the government to our overall budget would be approximately 55% or so, but during a games year, the figure goes anything from that 55 to up to about 70–80%. (Interview 17)

However, the government does not impose excessive strings to the grants for the BNOC, nor sets any targets for sports performance. Nevertheless, the annual budgetary process is a source of tensions. Government grants are given to the BNOC based on an annual draft budget request submitted to the Ministry of Sports. During that process, the Ministry of Sport, the Treasury or the Parliament might question some of the BNOC budget lines. It is in that process where personal relations with Permanent Secretaries, desk officers or even the Minister are important to iron out any problems, as recognized by interviewees from both sides.

#### Guatemala: Public funding for sport shrined in the constitution

The specific Guatemalan legal framework grants the GNOC a very significant fixed budget, which can be spent according to the budgetary provisions designed by the GNOC itself. Accordingly, most the GNOC budget (93%) results from the government's constitutional budgetary provision. The substantial financial resources allow the GNOC to play a very active role. However, the external auditing process is very detailed and exhaustive, which reflects Guatemala's efforts to mitigate corruption. The GNOC tries to act as model organization and pursues a ‘management by process’ approach (Interview 5).

#### Sri Lanka: Prioritizing financial independence from the government

In order to avoid the governmental interference related to resource dependencies, the NOC SL, especially under the presidency of Hemasiri Fernando (1997–2018), prioritized financial independence:

We are self-financed and not dependent of the government funding for our activities. We try to avoid as much as we can to get government money in our accounts, so for example now we ask them to organize and pay travel expenses for athletes directly, rather than giving the money to us to do the bookings (Interview 2).

Accordingly, NOC SL's budget is the smallest of the three countries under study in this article, and it is also the budget with least government funding (5%). NOC SL raises its own income through different commercial operations such as the commercial exploitation of sport facilities, and also receives important income from the IOC through Olympic Solidarity (Interview 2). The financial independence implies limited resources and restricts the activities of the NOC SL. The largest expenditure area is training and education, in which NOC SL sees itself as a pioneer in south Asia (Interview 13).

### Participation of the NOC in national sport policy-making

#### Botswana: Expansion of NOC activities but limited influence due to organizational rivalries

The Botswana National Sports Commission Act of 2014 shrines BNSC as key government agency in sport, but crucially it does not restrict the BNOC's remit. With the help of IOC grants, the BNOC has expanded its activities, which has resulted in organizational rivalries and duplication of efforts.

The Long-Term Athlete Development Framework (LTADF) is an ambitious initiative of the BNOC to improve athlete identification and development from an early age. Yet, according to the BNSC Act of 2014, sport development is a responsibility of the BNSC. According to BNOC representatives, the LTADF emerged simply as an opportunity thanks to Olympic Solidarity funding. External observers, however, suggested that the BNOC was motivated by the deficiencies of BNSC's existing athlete development programs.

Well, you know, the Commission is doing something very similar to that [the LTADF], but the problem with it is that I do not think they understand it. So, they will not take the athletes through the stages of a long term development program. They will have group of athletes who comes every weekend or two or whatever, to come and play, and they develop from that, you know […]. They try to identify talent from that. But it's not structured. (Interview 3)

The LTADF represent an ambitious and very detailed initiative:

This document outlines the principles, best practices and considerations for Botswana Sport Associations and other relevant sport organizations in Botswana, to design and implement scientifically sound and practical programs and activities for participants at all ages and levels, for productive and rewarding participation and competition in sport and physical activity in Botswana. (40).

The initiative aimed to bring together all stakeholders in Botswana sport and should have been implemented in collaboration with the BNSC. Although the project found ministerial support, the LTADF has never taken off due to organizational rivalries with the BNSC:

The long-term athlete development program, it's a brilliant program. But what I can tell you is we made a mistake of identifying it with an institution. That should not have been encouraged. It should not be owned by the NOC […] It should not be owned by Commission, either. It is a Botswana framework. Because if the country pays for that, at the end of the day, who are we celebrating? We are celebrating a Botswana athlete; we are not celebrating a Commission athlete or an NOC athlete. […] So, I will say that is how I perceive that it was not accepted, but I do not think anyone just rejected it (Inteview 7).

Another area of tensions between the BNOC and BNSC is the preparation of teams for international competitions. The Olympic Charter stipulates that the BNOC is responsible for selecting and registering teams for Olympic competitions. The BNSC has been traditionally responsible for international single sport competitions, such as world championships or African championships. Tensions have arisen after the Association of National Olympic Committees of Africa (ANOCA) took over the responsibility for organizing the African Games, which implied that the BNOC became responsible for sending and heading the Botswana delegation to the African Games causing strong tensions between BNOC and BNSC. Finally, BNSC and BNOC reached an understanding to collaborate in sending the Botswana team to the 2019 African Games hosted by Morocco:

Botswana National Olympic Committee (BNOC) chief executive officer Tuelo Serufho announced that the Ministry of Youth Empowerment, Sport and Culture Development is investing BWP 18 million (€1.4 million) before the team leaves for Morocco for the Games due to begin on August 19. The BNOC is also working together for the first time with Botswana National Sport Commission to coordinate the team for the Games scheduled to end on August 31. ‘For the first time, the Association of National Olympic Committees in Africa and African Union (AU) Sport Council are delivering the Games together’ Serufho told local newspaper Mmegi (41).

The episode illustrates the regular organizational tensions as well as their usual solution. Despite tensions and clear political and turf wars, both organizations maintain a working relationship and are normally able to achieve a working compromise for the sake of Botswana sport.

#### Guatemala – Strong and powerful influence of a strong NOC

While the legal framework grants the GNOC substantial influence on high-performance sport, the GNOC is able to shape wider sport policies (beyond high-performance sport) in a more comprehensive manner through its participation in the National Council for Physical Education and Recreation (*Consejo Nacional de la Educación F*í*sica y la Recreación* – CONADER). CONADER is an inter-institutional coordinating body established in the National Sports Act. CONADER is formed by five persons: The president of the Directorate General for Physical Education (*Dirección General de Educación F*í*sica*–DIGEF, a governmental department) representing the Physical Education System, the presidents of both GNOC and CDAG (non-governmental organizations), the Deputy Minister of Culture and Sport and a person appointed by the President of the Republic. CONADER represents the forum in which Guatemala sport policy and its strategic plans are designed and adopted:

The legislative framework defines CONADER as the coordinating institution in charge of proposing national sport policy […] Thus, we could be seen as the top authority in the sport system, but only to some extent because decisions need to be taken by consensus as they then need to be observed and implemented by those around the table (Interview 14)

Regardless of unanimity rule, GNOC and CDAG tend to dominate the discussions because they seem to be the institutions with a more elaborated strategic vision:

The current leaders of the GNOC and the CDAG have more experience and have been in their positions for longer. They bring a clear strategic vision and this is very positive for us as a system. The governmental side, however, experiences far more changes, so it is difficult for them. (Interview 14)

CONADER solidifies in this way the role of the GNOC as key actor in national sport policy-making communicating on equal footing with the government.

Since the NOCs are supposed to serve the diffusion of Olympic sports by influencing national education programs, the GNOC has also assumed a very proactive role as its Olympic Education Academy collaborates with different government departments:

Our mandate, according to the Olympic Charter, is to disseminate Olympic values through the population. Therefore, we are completely aware that having good relations with governmental departments will help us to achieve those objectives. We can be much more powerful and reach out to more people, especially around the country, that if we were just on our own. Therefore, as we seek to reach the general population, we make a conscious strategic decision to set up collaborations with governmental departments and ministries. I would say this is especially true in the area of education, but we seek to cooperate with any department that can help us […] (Interview 4)

The GNOC formalized these collaborations through the signature of a Framework Agreement with the Ministry of Education in 2018 for a period of 3 years. The Framework Agreement stipulated that collaboration was focused on design, funding, implementation, and evaluation of specific programmes.

#### Sri Lanka – NOC Self-restraint, political interference and instrumentalization

Sri Lanka's sport system is heavily politicized by means of government funding and regulations. This politicization is also reflected in personal and political ties between government and the sports sector (39). Thus, the former president of NOC SL, Hemasiri Fernando, was a Secretary in the Ministry of Postal Services, and Chef de Cabinet to the country's Prime Minister before being elected to chair the NOC. After the end of his tenure in 2018, Fernando was appointed as Defense Secretary. In such a heavily politicized setting, sport administrators inevitably end up tangled in complex networks of personal and political relationships. The collaboration between NOC SL and governmental departments depends either on good personal relations or the political capital of NOC SL leadership. The politicization of sport becomes evident in the selection of national teams to participate in international competitions and the bidding for sport event hosting.

Sri Lanka has implemented a very specific procedure for national team selection that gives very strong powers to the government, with the sports minister supervising and signing off the list of athletes for international competitions. This very interventionist policy is related to a historical incident. After two cricket selectors controversially picked themselves to play ahead of other cricketers for a 1968 tour of England, the tour was eventually canceled by ministerial intervention. The incident stirred major controversies and is also cited as a reason for the interventionist character of the 1973 Sports Law (42). Despite the time that has passed, current government officials justified the politicized selection process with the difficult legacy of the 30-year long civil war. Political control is supposed to prevent athletes from displaying symbols in support of the country's Tamil minority (Interview 10).

Hence, whereas the selection of athletes and teams is usually the privilege of national sport federations, in Sri Lanka the Sports Ministry appoints a National Selection Committee, comprised of Secretary General and President of the NOC, and three other “independent” members. For major competitions, the National Selection Committee scrutinizes the suitability of each individual athlete selected by sport federations. Eventually, the Sports Ministry has the power to veto athletes' selection, a power that has been used at times (Interview 2). The politicized selection process is a source of persistent conflict between government and NOC SL. Interviewees claimed that, in the past, some athletes employed political ties to get selected for international competitions (Interview 11), although this could not be verified independently.

The hosting of sports events also demonstrates the strained and complex relationship between government and NOC SL. Sri Lanka organized the South Asian Games twice (1991 and 2006) and the South Asian Beach Games once (2011), which were characterized as “small, manageable games that fit the limited capabilities of the country” (Interview 18). However, the government decided to bid for the Commonwealth Games in 2018, which qualify as a mega sport event. One interviewee described the bid as “purely a propaganda project” of the government. The NOC SL and the national sport federations had serious doubts about the project's feasibility and feared negative effects on Sri Lanka's developing economy. However, according to several interviewees, non-cooperation on behalf of the NOC SL “was not an option.” Due to the institutional framework of international sport, the NOC SL was inevitably a key actor in the project, which implied close cooperation with top government officials. The government funded the preparation of the bid, whereas the NOC SL provided its expertise in event organization. The bid was eventually lost to Australia, which many of the interviewees deemed as the more optimal outcome for Sri Lanka. This case, again, demonstrates how NOC SL had to follow political decisions even if these were considered to be negative for sport in the country and for the organization itself.

## Discussion and conclusion

In this article, we have argued that the IOC imposes sports autonomy–as a governance concept, which originated in the Global North–on countries from the Global South that have sought to be part of the Olympic Movement. However, in accordance with policy transfer research, we have tried to show that the coercive transfer of a governance transplant has its limits. With regard to sports autonomy, we have characterized political self-restraint and a strong civil society as crucial prerequisites for a successful transplantation. If these prerequisites are absent, the governance transplant is translated in accordance with path-dependent domestic institutions and practices. Translation means here that the transplant is adapted and customized to make it compatible with existing local political practices (22). Our case study evidence supports these ideas.

The full or unrestricted transfer of the sports autonomy *via* constitutional provisions in Guatemala, which could be characterized as “gold plating” from an IOC perspective, results from the fact that the country had a domestic legacy of sports autonomy. Accordingly, elite sport development is largely given into the hands of autonomous (non-governmental) organizations. In Guatemala, the GNOC is actually an agenda-setter for the government in sport policy-making. Accordingly, our case studies demonstrate also in case of a good “fit” with domestic legacies, the governance transplant can have very strong influences on domestic institutions and practices.

A note on the case of Guatemala, though. During the time that took the review of this article after its first submission, the GNOC has been involved in a major battle with the government of the country as a result of the latest GNOC elections. Actually, the IOC has suspended the membership of GNOC (43), which means that Guatemala is, for the moment, outside of the Olympic Movement and, if this were to be maintained, the country could not compete in the Olympic Games. The conflict refers to the latest GNOC presidential elections. The incumbent president, Gerardo Aguirre, sought re-election, but faced a competing candidate, thought to be supported by the government of the country (44, 45).

At the time of writing, the latest development is that Guatemala's Constitutional Court intervened in the legal dispute, finding in favor of the competing candidate (44). The GNOC has accused some Constitutional Court justices of being politically biased toward the government and not upholding the constitution's provisions on sports autonomy (46). The intervention of the Constitutional Court in the GNOC elections, and the whole legal process is vehemently opposed by the current GNOC leadership and its president, Gerardo Aguirre, who accuse the country's government of willing to control federated sport and the GNOC through the back door (46). In response to this, the IOC (43) decided to suspend Guatemala considering that the interventions of both the government and the country's Constitutional Court were a breach of the fundamental principle of autonomy. Membership suspension is the IOC's measure of last resort to protect the autonomy of sport, and it is often used to protect NOCs from political intervention of governments [(34); see also (9)]. In a way, these latest developments could question some of the conclusions from our research in relation to the strong autonomy of the GNOC, and how the institutional framework of the country empowers the GNOC in the Guatemala sport system. However, it can also be argued that, actually, the government of Guatemala finds so difficult to politically interfere in the GNOC that it had to resort to extreme and very controversial measures, such as intervening in the GNOC electoral process. Furthermore, even in this case, it is not being easy for the government to influence the electoral process because the IOC and the NOC have still tools to enforce autonomy. At the end of the day, despite the efforts, Gerardo Aguirre still considers himself to be the GNOC president at the time of submitting this article, even if the country has seen its IOC membership suspended and the rival candidate has declared himself to be the rightful president of the GNOC. On the other hand, this could also be seen as a test whereby the GNOC and its president, Gerardo Aguirre, are testing how autonomous the GNOC is, and how far they can go with exerting political influence in the country's sport system (45), even when facing heavily interventionist impulses from the government. For our purpose in this article, it was necessary to mention these latest developments for the sake of transparency, but also to reflect on the need for further research.

Whereas these latest developments might introduce some nuances, they do not challenge fundamentally one of our main conclusions, though: the stark difference in the degree of autonomy between the case of the GNOC and the NOCs of Botswana and Sri Lanka. Indeed, without the IOC stipulations on sports autonomy, the NOCs of Botswana and Sri Lanka would very likely be part of a state controlled and/or strongly politicized sport system. Notwithstanding the acceptance of the transplant (with different forms of translation in these countries), persistent misfits are visible. In Botswana, a stable democracy, sports autonomy contradicts the government's ambition to play an active role in domestic sport policy-making. Thus, tensions between the government's sport agency and the autonomous NOC occur regularly although they are usually solved on the base of a shared commitment to national sport development. In contrast, in Sri Lanka's politicized post-civil war society, the idea of sports autonomy does not figure, which results in self-restraint and marginalization of the NOC. There is little evidence for a better integration of the governance transplant into domestic sport policy-making (Table 4).

**Table 4:** The autonomy of the National Olympic Committees.

	**Botswana**	**Guatemala**	**Sri Lanka**
Domestic context and legacies	Strong government ambitions in sport policy-making	Strong legacy of sport autonomy	Strong interventionist legacy, politicization due to civil war
Formal compliance	Formal acceptance of autonomy but unclear division of responsibility between government agency and NOC	Sports autonomy adopted in constitution	Legal framework does not respect sports autonomy
Provision of public funding	Strong dependence on government funding but limited government interference in budgeting	Fixed provision of public funding specified in the constitution, autonomous budgeting by the NOC	Prioritization of financial independence in order to avoid political interference
Participation in sport policy-making	Expansion of NOC activities but limited influence due to organizational rivalries	Strong influence of NOC	Self-restraint, political interference and instrumentalization
Translation of the governance transplant	Iron out tension	Full transfer or “gold-plating”	Marginalization

The case of Sri Lanka comes also with broader implications as it raises the question why IOC practices a rather conciliatory approach to the enforcement of sports autonomy, which differs very much from the one adopted by the *Fédération Internationale de Football Association* (FIFA) (8, 47), or actually the more recent decision on Guatemala. Even though Sri Lanka failed to amend its domestic legal framework in accordance with the IOC's stipulation on sports autonomy, Sri Lanka got not suspended from Olympic competitions. We claim that the conciliatory approach is indicative of the IOC's fundamental enforcement dilemma. While the IOC controls the access to the Olympics, the Olympic Movement's long-term viability in political, economic and social terms rests on the successful diffusion of its specific sports model, which includes cooperation with national governments around the world. The difference with football and FIFA is that, while football migrated with apparent ease all over the world due to its simple rules and low infrastructure needs, diffusing Olympic sports requires support and investments by public authorities in different policy domains. Moreover, these requirements can change as the recent modernization efforts of the IOC show. The IOC aims to increase the attractiveness of the Olympic Games for younger audiences by including formerly non-Olympic youth sports, while getting rid of traditional sports, which no longer appeal to audiences. Such changes imply that public authorities face some uncertainty whether their investments will pay off. Hence, in some respects, the IOC faces similar enforcement problems as other transnational regulators, which operate in diverse national settings and remain dependent on public authorities (10). Strict enforcement of a governance transplant might risk losing the support of public authorities and thus contradict the diffusion aims of the IOC. Actually, the IOC has greatly modulated its emphasis on sports autonomy lately (9). The departure from strict enforcement of sports autonomy indicates that the IOC is aware of its dependence on public authorities in diverse political and social contexts.

The broader theoretical relevance of the fate of sports autonomy in the Global South is that international sport represents a transnational policy domain where asymmetrical transfer dynamics from the Global North to the Global South might come to an end. The political and organizational center of the Olympic Movement has moved from the West toward Asia, Middle East and Eastern Europe. As governance transplant, which is likely to face substantial misfits with national legacies and political ambitions, sports autonomy might be ultimately at stake (28). Hence, in the not-too-distant future, the Global South might use its increasing influence in international sports to get rid of the obligation to accept governance transplants in exchange for market access.

However, before making bold claims about the future of sports autonomy, it is important to emphasize the limitations of our comparative case study design. Our small sample size does not allow to fully explore potential sources of misfit between the governance transplant and local path-dependences and configurations of formal and informal institutions. Future research on sports autonomy as governance transplant should cover more cases sampled on the base of a larger set of potential explanatory variables.

## Data availability statement

The datasets presented in this article are not readily available because the raw and anonymized data supporting this article can only be made available upon express authorization of the participants. Requests to access the datasets should be directed to the corresponding author.

## Ethics statement

This study, involving human participants, was reviewed and approved by Loughborough University Ethics Review Sub-committee. The participants provided their written informed consent to participate in this study.

## Author contributions

BG undertook data collection in the three fieldwork visits. Both authors contributed to conception and design of the study, and to the thematic analysis of the coded data; they contributed equally to drafting and editing the manuscript, and to manuscript revision. Both authors read, and approved the submitted version.

## Funding

Research presented in this article was supported by the International Olympic Committee [Advanced Olympic Research Grant Programme - 2018/2].

## Conflict of interest

The authors declare that the research was conducted in the absence of any commercial or financial relationships that could be construed as a potential conflict of interest.

## Publisher's note

All claims expressed in this article are solely those of the authors and do not necessarily represent those of their affiliated organizations, or those of the publisher, the editors and the reviewers. Any product that may be evaluated in this article, or claim that may be made by its manufacturer, is not guaranteed or endorsed by the publisher.
